# Affinity and Structural Analysis of the U1A RNA Recognition Motif with Engineered Methionines to Improve Experimental Phasing

**DOI:** 10.3390/cryst11030273

**Published:** 2021-03-10

**Authors:** Yoshita Srivastava, Rachel Bonn-Breach, Sai Shashank Chavali, Geoffrey M. Lippa, Jermaine L. Jenkins, Joseph E. Wedekind

**Affiliations:** 1 Department of Biochemistry & Biophysics and Center for RNA Biology, University of Rochester School of Medicine & Dentistry, Rochester, NY 14642, USA; 2 Structural Biology and Biophysics Facility, University of Rochester School of Medicine & Dentistry, Rochester, NY 14642, USA; 3 Present address: Division of Biology, Alfred University, Alfred, NY 14802, USA

**Keywords:** RNA-protein interactions, RNA crystallization, isothermal titration calorimetry, X-ray crystallography, selenomethionine, anomalous diffraction, U1A RRM, S–π interaction

## Abstract

RNA plays a central role in all organisms and can fold into complex structures to orchestrate function. Visualization of such structures often requires crystallization, which can be a bottleneck in the structure-determination process. To promote crystallization, an RNA-recognition motif (RRM) of the U1A spliceosomal protein has been co-opted as a crystallization module. Specifically, the U1-snRNA hairpin II (hpII) single-stranded loop recognized by U1A can be transplanted into an RNA target to promote crystal contacts and to attain phase information via molecular replacement or anomalous diffraction methods using selenomethionine. Herein, we produced the F37M/F77M mutant of U1A to augment the phasing capability of this powerful crystallization module. Selenomethionine-substituted U1A(F37M/F77M) retains high affinity for hpII (*K*_D_ of 59.7 ± 11.4 nM). The 2.20 Å resolution crystal structure reveals that the mutated sidechains make new S-π interactions in the hydrophobic core and are useful for single-wavelength anomalous diffraction. Crystals were also attained of U1A(F37M/F77M) in complex with a bacterial preQ_1_-II riboswitch. The F34M/F37M/F77M mutant was introduced similarly into a lab-evolved U1A variant (TBP6.9) that recognizes the internal bulged loop of HIV-1 TAR RNA. We envision that this short RNA sequence can be placed into non-essential duplex regions to promote crystallization and phasing of target RNAs. We show that selenomethionine-substituted TBP6.9(F34M/F37M/F77M) binds a TAR variant wherein the apical loop was replaced with a GNRA tetraloop (*K*_D_ of 69.8 ± 2.9 nM), laying the groundwork for use of TBP6.9(F34M/F37M/F77M) as a crystallization module. These new tools are available to the research community.

## Graphical Abstract

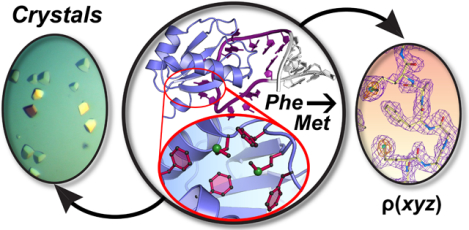

## 1. Introduction

RNA is integral to cell function and has overturned old rules that erroneously credited proteins as master regulators of key biological functions [1]. Unlike the human proteome, which arises from only a small fraction of the DNA blueprint (0.05%), most of the genome is transcribed into RNA [2,3]. Accordingly, many RNA transcripts are crucial molecules that function to guide DNA synthesis, as guardians against invading nucleic acids, and as controllers of gene regulation at the transcriptional and translational levels [1]. The importance of RNA is heighted by the fact that only a small fraction of the proteome (~3.5–10%) is likely druggable [4,5]. Consequently, RNA has been viewed increasingly as a therapeutic target [6–8].

On the road to understanding RNA molecular function, near atomic-level structures of several important RNA classes have been elucidated using experimental approaches [9–22]. This work has demonstrated that RNA can adopt elegant three-dimensional folds with distinct topologies and recurrent architectural motifs [23–33]. Many of these RNAs include cavities and deep grooves poised to receive natural or artificial ligands [34]. At present, the preponderance of known RNA structures has been derived from x-ray crystallography. Albeit, successful RNA crystallization can be a rocky pathway that often represents the rate-limiting step of a structure determination. An ingenious strategy that has facilitated the success of this process has been the use of RNA binding proteins to promote crystallization [35]. In this respect, U1A RNA-recognition motif 1 (RRM1) has shown considerable efficacy (Table 1). The main strategy has been documented.

**Figure 1.**
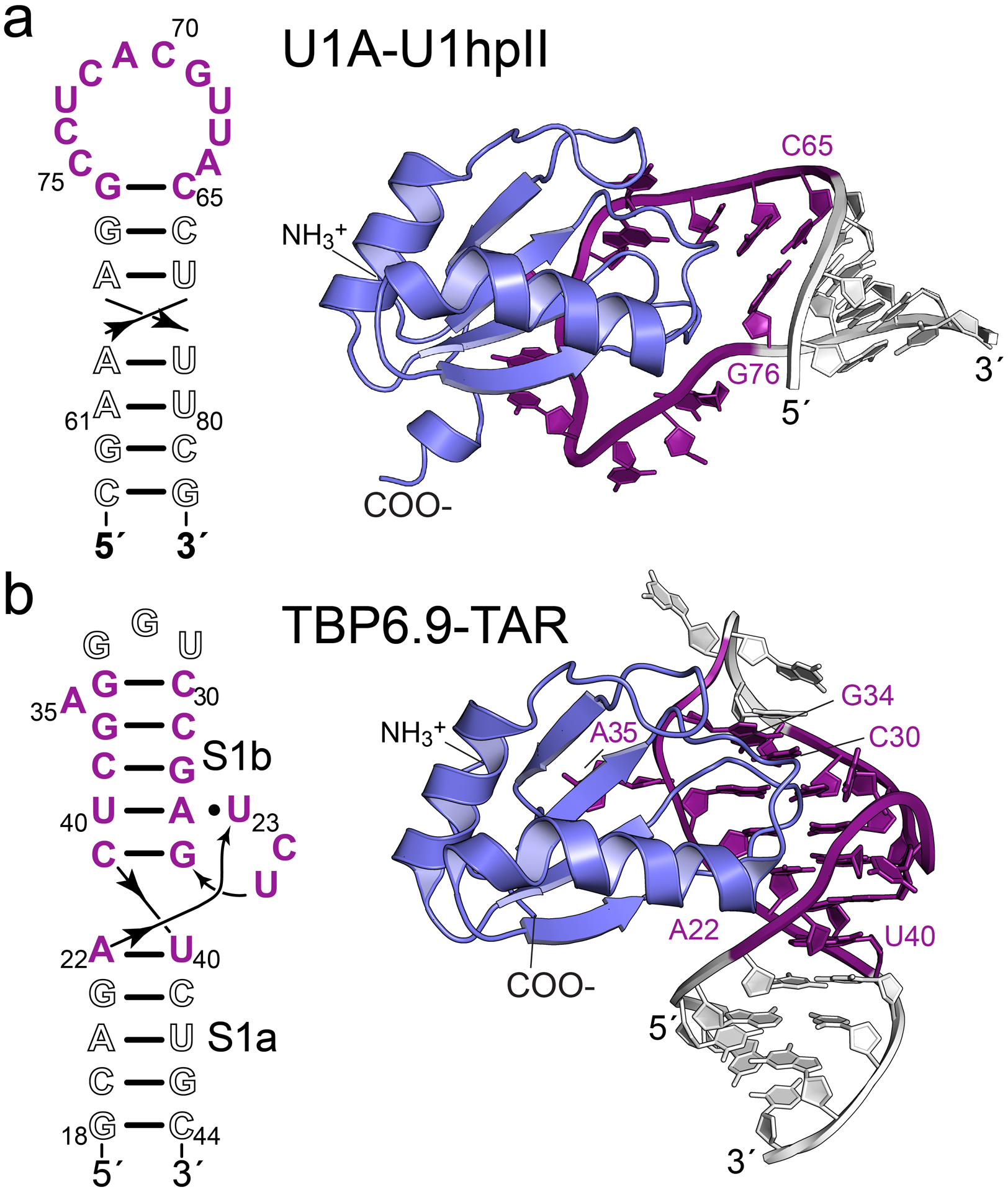
Schematic and cartoon diagrams of RNA sequences targeted by U1A and the related HIV TAR-binding protein (TBP) variant 6.9. (**a**) (Left) Human U1A RRM1 binds the short hairpin II (hpII) sequence of U1 snRNA. Binding occurs primarily in the single-stranded loop region closed by the canonical C65-G76 base pair. This region is necessary and sufficient for U1A binding. (*Right*) Cartoon drawing of the double-mutant protein (dm)U1A-hpII 21-mer co-crystal structure [56], emphasizing protein recognition of the hpII loop (PDB entry 1urn); the duplex region (white) is not involved in hpII binding and can be replaced by a target sequence to promote RNA crystallization. (**b**) (Left) Lab-evolved TBP6.9 recognizes TAR RNA at the internal bulged loop, which includes a U23 A27-U40 base triple and bulged A35 within helical stem S1b [51,52]. An arginine fork is a key determinant of recognition at the G26 major-groove edge and the U23 backbone [59]. (*Right*) Cartoon drawing of the TBP6.9-TAR co-crystal structure [52], emphasizing protein recognition of the TAR internal bulged loop (PDB entry 5xh0); the apical loop and S1a (white) are not involved in TAR binding and are hypothesized herein to allow replacement within a duplex target sequence to promote RNA crystallization and phasing.

Elsewhere in a series of thought-provoking reviews that outline practical methodology [53–55]. The essence of the approach is that the hairpin II (hpII) loop of the U1-snRNA is substituted into a non-conserved loop position of a target RNA (Figure 1a). U1A RRM1 variant Y31H/Q36R (i.e., double-mutant protein or dmU1A) is then added to promote crystallization [56,57]. In the context of the RNA target, the dmU1A variant can provide new protein-protein and protein-RNA contacts that favor formation of a well-ordered crystal lattice. A second function of the dmU1A variant is to serve as a phasing module [53–55]. The known dmU1A-hpII complex can serve as a partial search model for molecular replacement (MR). Alternatively, the five methionine residues of dmU1A can be substituted with selenomethionine for single-wavelength anomalous diffraction (SAD) or multiwavelength anomalous diffraction (MAD) phasing [53–55]. Both SAD and MAD take advantage of resonant x-ray interactions with selenium K-shell electrons, as reviewed elsewhere [58].

Under optimal circumstances, one selenium is sufficient to phase a scattering mass of approximately 75–100 amino acids [60,61], which is equivalent to ~22–33 nucleotides of RNA in terms of scattering strength [62]. To improve the signal available for de novo dmU1A-mediated selenomethionine phasing, we undertook a mutational and RNA binding analysis of dmU1A with the goal of adding additional ordered methionines for experimental, selenium-based phasing. Here we describe the bacterial expression, purification, and affinity analysis of the dmU1A(F37M/F77M) mutant wherein phenylalanine residues were changed to methionine. An RNA binding analysis indicated that the mutated, selenomethionine-labeled protein retains tight binding to hpII, and readily crystallizes in an isolated form, in complex with hpII RNA and in complex with a class II preQ_1_ riboswitch from *Lactobacillus rhamnosus* [63]. The crystal structure of the dmU1A(F37M/F77M) variant is described and compared to other dmU1A molecules crystallized in the absence and presence of RNA. A SAD phasing analysis demonstrates that the new Se atoms contribute useful anomalous signals to phasing. In addition, we present evidence that lab-evolved TAR-binding protein variant 6.9 (TBP6.9) derived from dmU1A retains HIV-1 TAR RNA binding when three core phenylalanines are mutated to methionine. This TBP6.9(F34M/F37M/F77M) variant recognizes the 16-nucleotide internal bulged loop of TAR RNA but does not require the apical loop or stem 1a for target RNA binding (Figure 1b). Indeed, our observations herein confirm that TBP6.9(F34M/F37M/F77M) strongly binds the TAR internal bulge in the absence of the apical loop. These observations lay the groundwork for use of TBP6.9(F34M/F37M/F77M) as a crystallization and phasing module when the target RNA is not amenable to integration of a hpII RNA loop. Although a comprehensive analysis of crystallization and phasing awaits future studies, we have made our protein variants available to researchers seeking immediate access to new tools for crystallization and experimental phasing of RNA.

## 2. Experimental Procedures

### 2.1. Expression of Se-Met-labeled dmU1A(F37M/F77M) and TBP6.9(F34M/F37M/F77M)

Synthetic genes for dmU1A, dmU1A(F37M/F77M), and TBP6.9(F34M/F37M/F77M) were produced by DNA synthesis and subcloned into pET28a (GenScript Inc.). Each construct has a tobacco etch virus (TEV) protease cleavage site in place of the thrombin proteolytic site. The Y31H/Q36R mutation that defines the dmU1A variant was integrated into each sequence to promote crystallization [56,57]. Expression of methionine-containing dmU1A and TBP6.9 was described as reported [52]. Selenomethionine labeling of dmU1A(F37M/F77M) and TBP6.9(F34M/F37M/F77M) was performed essentially as described [64]. Each plasmid was transformed into *E. coli* BL21(DE3) cells (Novagen) for expression. Bacteria were streaked onto Luria–Bertani (LB) agar plates containing 50 μg mL^−1^ kanamycin and grown at 37 °C. Single colonies were used to inoculate cultures of LB broth for overnight growth. A volume of 60 mL was used for each liter of expression media. The cells were pelleted by centrifugation for 10 min at 2880 *x*g and the LB media was decanted. Cells were suspended in 10 mL of minimal media (MM) containing 1x M9 salts (Na_2_HPO_4_, KH_2_PO_4_, NaCl, and NH_4_Cl), 2 mM MgSO_4_, 0.1 mM CaCl_2_, 0.5% (*w/v*) glucose, 2 mg L^−1^ biotin, 2 mg L^−1^ thiamine, and 1x MEM vitamin mix (Thermo Fisher Scientific). The cell suspension was transferred into a larger 1 L volume of MM within a baffled 3 L Fernbach flask sealed with cheesecloth. Cells were grown to an optical density at 600 nm of 0.4, followed by addition of an amino acid mixture (100 mg L^−1^ of Lys/Phe/Thr and 50 mg L^−1^ of Iso/Leu/Val) to repress methionine synthesis, and selenomethionine (Sigma–Aldrich) (50 mg L^−1^). After reaching an OD at 600 nm of 0.6 the cells were cooled to 20 °C, induced with 0.5 mM isopropyl-β-D-thiogalactoside (IPTG), and expressed for 20 h. The cells were pelleted by centrifugation at 2880*x*g and frozen in N_2_ (*l*).

### 2.2. Purification of dmU1A, TBP6.9, dmU1A(F37M/F77M), and TBP6.9(F34M/F37M/F77M)

Cells were thawed in a cell-lysis buffer (CLB) containing: 0.05 M Na-HEPES pH 8.0, 0.3 M NaCl, 0.02 M imidazole pH 8.0, 0.0005 M EDTA, 0.005 M β-mercaptoethanol (β-ME) and 0.01% (*v/v*) Brij35; the cell slurry was made 2 mg mL^−1^ in hen egg white lysozyme (VWR). After 20 min, cells were sonicated with a Sonic Dismembrator 60 (Fisher Scientific) and treated with 100 μg mL^−1^ DNase and RNase (Roche). The clarified supernatant was bound in batch to Ni-NTA resin (Pierce) equilibrated with CLB. After 2 h of nutation at 4 °C, resin was poured into a 1.5 cm *×* 10 cm gravity-flow column (CrystalCruz), washed with 40 column volumes of CLB and 5 column volumes of wash buffer (WB) comprising: 0.05 M Na-HEPES pH 8.0, 0.15 M NaCl, 0.04 M imidazole pH 7.5, 0.005 M EDTA, 0.005 M β-ME and 0.01% (*v/v*) Brij35. Fractions were collected in 3 mL volumes using an elution buffer (EB) comprising: 0.05 M Na-HEPES pH 8.0, 0.15 M NaCl, 0.2 M imidazole pH 7.5, 0.005 M EDTA, 0.005 M β-ME and 0.01% (*v/v*) Brij35. Fractions were pooled based on absorption at 280 nm and diluted with WB without imidazole to a achieve a final imidazole concentration <0.03 M. The 6xHis-TEV protease [65] was added (1:100 TEV:protein) and the mixture was incubated at 4 °C to remove the 6His tag. After 16 h the reaction was incubated in batch with pre-equilibrated Ni-NTA to retain the uncut protein and TEV; the supernatant was decanted and saved. The protein was loaded at 0.5 mL min^−1^ onto a 5 mL HiTrap SP FF column (Cytiva) using an ÄKTA Pure (Cytiva). Samples of dmU1A and dmU1A(F37M/F77M) were washed and eluted using a linear gradient comprising: 0.15 to 0.85 M NaCl, 0.05 M Na-HEPES pH 8.0, 0.0005 M EDTA, and 0.005 M β-ME. The proteins eluted around 50–70 min corresponding to 50–70% of the maximum salt concentration. TBP6.9 and TBP6.9(F34M/F37M/F77M) were purified similarly except that a steeper salt gradient of 0.15 to 1.5 M NaCl was required for the HiTrap SP FF column to reduce non-specific RNA interactions caused by the arginine-rich content of the β2–β3 loop; other minor changes were made to the buffers as described [52]. Each concentrated protein sample (~150 μm) was subjected to size exclusion on a HiPrep (16/60) Sephacryl S-300 HR column (Cytiva), except TBP6.9(F34M/F37M/F77M), which was sufficiently pure after ion exchange for isothermal titration calorimetry (ITC) analysis. Molecular weight standards were obtained as a kit (Cytiva). Samples of dmU1A (MW 11.4 kDa) and dmU1A(F37M/F77M) (MW 11.5 kDa) each eluted with retention times consistent with monomeric subunits (Supplementary Figure S1). TBP6.9 (MW 11.5 kDa) exhibited longer chromatographic retention than predicted by mass, eluting at or >1 column volume (data not shown). Protein purity was estimated at > 95% by Coomassie-stained SDS-PAGE. The yield from LB media was 2 to 3 mg L^−1^ of cells; the yield from MM was ~1 mg L^−1^ of cells.

### 2.3. ITC Analysis of dmU1A, dmU1A(F37M/F77M), TBP6.9, and TBP6.9 (F34M/F37M/F77M)

The hpII 24-mer and GNRA-TAR 30-mer strands were made by chemical synthesis (Horizon Discovery). The HIV TAR 27-mer and *Lactobacillus rhamnosus* preQ_1_-II riboswitch 84-mer strands were produced by *in vitro* transcription as described [66]. The sequences were: 5´-GGAGAUCUGAGCCUGGGAGCUCUCUCC for TAR and 5´-GGAAGGCCAUUGCACUCCGGUCUUCCACGACGAUACUUACUUUCCUUUGAUCGUCGUUACUGGCUUCGGCCACAAAGGAGA for the riboswitch. Each RNA strand was purified by denaturing gel electrophoresis, desalted and lyophilized as described [30,66]. For ITC, each RNA strand was dissolved in 0.01 M Na-HEPES pH 7.5 and heated at 65 °C. After 3 min, ITC buffer pre-heated to 65 °C (0.05 M Na-HEPES pH 7.5, 0.05 M NaCl, 0.05 M KCl, and 0.002 M MgCl_2_) was pipetted into the RNA, followed by a 5 min incubation at 65 °C; the sample was cooled overnight to room temperature. Each sample was dialyzed at 4 °C overnight against 4 L of ITC buffer containing 0.005 M β-ME. To attain a buffer match, each pre-concentrated protein sample was co-dialyzed in the same reservoir as the RNA. Protein samples were diluted in used dialysis buffer to concentrations ~10-fold higher than the RNA (7–10 μM). ITC measurements were conducted at 20 °C using a PEAQ-ITC (Malvern Panalytical) with protein in the syringe and RNA in the cell [51,52,67]. The time between injections was 150 sec with a total of 19 injections. Thermograms were analyzed with PEAQ-ITC Analysis software using a 1:1 binding model (Supplementary Methods). This model was supported by the stoichiometry (n) of binding (i.e., the ligand–receptor ratio), which produced values close to unity. Other models were tested, including stoichiometries of 2:1, to assess sequential or two sets of binding sites (e.g., resulting from cooperativity). However, these fits were rejected due to very poor fits of the thermogram data to binding isotherms based on χ^2^ values and visual inspection. For dmU1A, the 1:1 model yielded χ^2^ values of 0.3, whereas the two-site and sequential-binding models produced χ^2^ values of 4.0 and 159. Similarly, selenomethionine-labeled dmU1A(F37M/F77M) produced χ^2^ values of 0.02, whereas the two-site and sequential-binding models produced χ^2^ values of 5.0 and 55. As a control for protein oligomerization, dmU1A and selenomethionine-labeled dmU1A(F37M/F77M) were injected into buffer alone. No significant heats of dilution were detected (Supplementary Figure 2), confirming that there is no appreciable dimer-monomer equilibrium [68]. The monomeric states of these samples at concentrations used for ITC were confirmed further by size-exclusion chromatography (Section 2.3). Formation of RNA-protein complexes in 1:1 ratios is consistent with known co-crystal structures of the parent complexes (Figure 1). For quality control, we maintained c values of ITC experiments in the preferred range of 10 ≤ c ≤ 500, which is necessary to attain reasonable fits of the equilibrium association constant, *K*_a_, from the binding isotherm [69]; here, c = n*K*_a_[M_T_], where [M_T_] is the receptor concentration in the cell [69]. We note titration of TBP6.9 into TAR slightly exceeded 1000 (Figure 3c). A c-value of 1000 is acceptable here because the fits were restrained by well-known concentrations of ligand and receptor, as well as the known 1:1 ligand-to-receptor stoichiometry [70]. ITC experiments were conducted in duplicate or triplicate.

### 2.4. Crystallization, x-ray Diffraction Analysis, and Structure Determination of dmU1A(F37M/F77M)

Pure selenomethionine-labelled dmU1A(F37M/F77M) protein was concentrated in a buffer comprising 0.050 M HEPES pH 8.0, 0.15 M NaCl, 0.005 M β-ME, and 0.0005 M EDTA to 5–8 mg mL^−1^ based on OD at 280 nm measured using a Nanodrop spectrophotometer (Thermo Scientific) using an extinction coefficient of 0.3319 (mg/mL)^−1^ cm^−1^. The protein was subjected to crystallization trials using hanging-drop vapor-diffusion at 20 °C [71] using an in-house incomplete factor screen [66] set up in two 24-well VDX plates (Hampton Research). A volume of 2 μL of precipitating agent was added to an equal volume of protein on siliconized glass cover slides (Hampton Research). The best single crystals were attained from 2.7–3.1 M sodium-acetate in a pH range of 6.5 to 7.0. Crystals were cryoprotected in 3.0 M Na-acetate pH 7.0 supplemented with 5% (*v/v*) glycerol. Crystals were flash cooled in thin nylon loops mounted on Crystal Cap Copper magnetic pins (Hampton Research) using a stream of N_2_ gas at 100 K produced by a 700 Series Cryostream (Oxford Cryosystems, Ltd). A complete data set was recorded using an X8 Prospector Ultra microfocus IμS sealed-tube X-ray source equipped with an APEX II detector (Bruker AXS). A strategy was chosen to record the anomalous diffraction data using a combination of φ and ω scans. X-ray diffraction data were reduced in the Proteum software package keeping output anomalous pairs separate. The structure was determined by molecular replacement using Phaser [72] implemented in Phenix [73]. The dmU1A coordinates from PDB entry 1urn [56] were employed as a search model. Visualization of electron-density maps and manual model building were conducted in Coot [74]. Additional SAD phasing experiments were conducted using HySS, Phaser EP, Resolve and autobuilding as implemented in Phenix [72,73,75–77]. All cartoons and schematic diagrams derived from coordinates were generated using PyMOL (Schrödinger, LLC). Least-squares superpositions were performed in CCP4 using Lsqkab [78,79].

### 2.5. Co-crystallization of Se-Met Labelled dmU1A(F37M/F77M) with Hpii and Riboswitch RNA

The dmU1A(F37M/F77M) protein in complex with hpII RNA was crystallized as follows. A volume of 2 L of hairpin folding buffer (HFB) was prepared comprising: 0.010 M Na-HEPES pH 7.5, 0.05 M NaCl and 0.002 M MgCl_2_. Pure dmU1A(F37M/F77M) was dialyzed against ~2 L of HFB containing 0.005 M β-ME overnight at 4 °C. A 5 mL volume of HFB was heated to 65 °C in a thermostatted water bath. The synthetic 21-mer hpII RNA (Horizon Discovery), described previously [56], was purified as described in Section 4.3. The lyophilized RNA was dissolved in 0.010 M HEPES pH 7.5 to 0.40 mM and heated at 65 °C for 3 min. The RNA was then diluted slowly with 65 °C HFB to 0.040 mM. The dilute 21-mer solution was incubated at 65 °C for 5 min before turning off power to the water bath, allowing the RNA to cool slowly to room temperature overnight. Freshly dialyzed dmU1A(F37M/F77M) at a concentration of 0.04 mM U1A was added dropwise into the folded hpII RNA with gentle vortexing to give a 1:1 molar ratio. The complex was incubated for 30 min at room temperature. The protein-RNA complex was then concentrated using a Pierce 10 kDa MWCO 15 mL concentrator (Thermo Fisher Scientific) pre-washed with HFB. The sample was concentrated to 10 to 12 mg mL^−1^ (0.55 to 0.65 mM) at 20 °C to final volume of 50 μL. The complex was crystallized by the hanging-drop vapor-diffusion method [71] using 96 well plates. Screens were established using a Mosquito liquid-handling robot (SPT Labtech) in which 0.5 μL of precipitating agent from a Nuc-Pro HT screen (Jena Bioscience) was added to an equal volume of protein-RNA complex.

The dmU1A(F37M/F77M) protein in complex with an 84-mer preQ_1_-II riboswitch containing the hpII binding site in P1 was crystallized as follows. A volume of 10 mL of riboswitch folding buffer with preQ_1_ (RFBQ) was made from 0.010 M Na-cacodylate pH 7.0, 0.005 M MgCl_2_ and 0.0015 M preQ_1_. A volume of 5 mL of RFBQ was heated to 65 °C in a thermostatted water bath. A small volume of lyophilized riboswitch RNA (50 μL) stored at −20 °C was dissolved in 0.01 M Na-cacodylate pH 7.0 to 0.5 mM and heated for 2 min at 65 °C. The riboswitch was diluted slowly with 65 °C RFBQ to a final volume of ~5 mL. The solution was incubated at 65 °C for 3 min before turning off the water bath to allow overnight cooling to room temperature. The dmU1A(F37M/F77M) sample freshly dialyzed against HFB containing 0.005 M β-ME was diluted to 0.04 mM. The protein was added dropwise into the folded riboswitch with gentle vortexing to give a 1:1.2 molar ratio. The complex was incubated for 30 min at room temperature. The complex was concentrated as described above to ~10–12 mg mL^−1^. The protein was subjected to crystallization trials using hanging-drop vapor-diffusion at 20 °C [71] using an in-house screen [66], as described in Section 4.4. The best single crystals were observed in high salt and were optimized in 1.6 M Li_2_SO_4_, 0.050 M Tris-HCl pH 8.5, and 0.060–0.080 M MgSO_4_.

All crystals were tested initially for mechanical fragility by probing with a cat whisker mounted on a 27-guage needle with beeswax or by pressure from a stainless-steel acupuncture needle. Consistent with their high solvent content, our crystals were soft and easily crushed by gentle contact, as noted previously for biomacromolecules [80].

### 2.6. X-ray Diffraction Analysis of dmU1A(F37M/F77M) in Complex with a preQ_1_-II Riboswitch

Co-crystals of dmU1A(F37M/F77M) in complex with the *Lactobacillus rhamnosus* preQ_1_-II riboswitch 84-mer were cryoprotected by passing crystals through a 1:1 mixture of Parabar 10,312 and silicone oils (Hampton Research) before plunging into N_2_(*l*), as described [81]. Diffraction data were recorded at the Cornell High Energy Synchrotron Source (Ithaca, NY) on beamline A1. Due to the small crystal size, the beam was outfitted with capillary optics to generate an ~20 μm micro focus spot. X-ray diffraction data were collected on Q-210 CCD detector (ADSC Inc) at a distance of 300 mm. Data were recorded as 0.5° φ-rotations with an exposure time of 30 sec per image. Data were reduced using HKL2000 (HKL Research, Inc) [82]. Representative intensity statistics are provided in Supplementary Table S1.

## 3. Results and Discussion

### 3.1. Rationale of Phenylalanine-to-methionine Mutants in the dmU1A Hydrophobic Core

**Figure 2.**
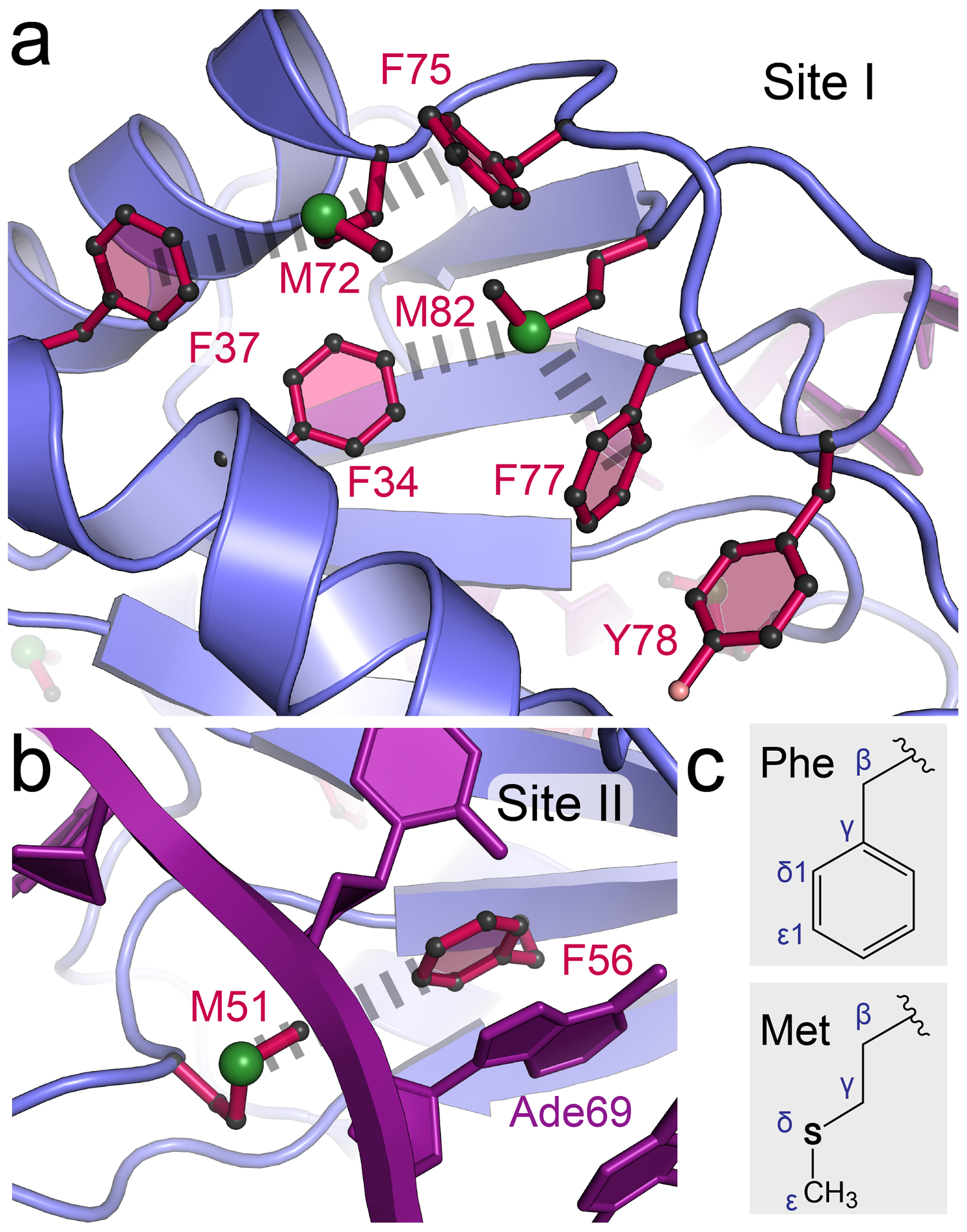
Rationale for Phe-to-Met mutations in U1A based on S–π interactions in the hydrophobic core. (**a**) Close-up view of the ‘site I’ hydrophobic core depicting aromatic and methionine residues. Hash marks indicate putative S-π interactions between specific Phe and Met residues. Here and in panel **b**, protein coordinates were derived from PDB entry 1urn, referred to as dmU1A, due to the double-mutations made to the protein for crystallization [56,57]. (**b**) Close-up view of the site II hydrophobic core on the side of the β-sheet opposite site I. F56 interacts directly with M51 and Ade69 of hpII. (**c**) Drawing of the phenylalanine and methionine sidechains to compare their relative sizes and atomic compositions.

First, we considered where to add additional methionines and which residues to mutate. Although frequently encountered at the N-terminus of proteins due to its role as the genetic signal to start translation, methionine is non-polar making it well suited to occupy the hydrophobic cores of globular proteins. Indeed, analysis of T4 lysozyme has suggested that leucine is a first-choice site for methionine substitution in the hydrophobic core along with other residues in the series L > F > I > V [83]. Accordingly, we sought to identify non-methionine residues within dmU1A that would maintain core packing but with sufficient volume to accommodate selenomethionine, which exhibits a slightly larger van der Waals radius than sulfur (i.e., 1.85 Å versus 2.00 Å) and a longer covalent bond (1.80 Å versus 1.95 Å) [64,83]. We noted that dmU1A contains three well-ordered methionines in its hydrophobic core located above and below each face of the four-stranded β-sheet. Specifically, M72 and M82 are positioned on the face that opposes the hpII RNA binding site, designated site I for convenience here (Figure 2a). M72 and M82 pack against several β-branched amino acids including I12, I14, L19, L26, Leu 30, and I84, as well as aromatic amino acids F34, F37, F75, F77, and Y78. The opposite β-sheet face contains M51, which packs against F56 (Site II) part of the conserved RNP1 motif [56] that recognizes hpII RNA by stacking with Ade69 (Figure 2b). Due to the central role of site II in RNA recognition, we chose to avoid mutations in this region and opted instead for methionine insertion at site I.

Of the site I amino acids, phenylalanine seemed like the best choice for methionine substitution. The aromatic sidechain comprises Cβ, Cγ1, Cδ1, and Cε1 atoms that are analogous to the Cβ, Cγ, Sδ, and Cε atoms of methionine (Figure 2c). Although not aromatic, methionine has a hydrophobic alkyl chain and a thioether moiety that can engage in S–π interactions that can be more stabilizing than hydrophobic interactions by +1 to +1.5 kcal mol^−1^ [84]. Prior S–π analysis showed sulfur to be located above or in-plane with aromatic rings at distances ≤ 6.0 Å [84,85]. For dmU1A, site I is characterized by two in-plane S-π interactions from M72 to F37 and M82 to F34 (Figure 2a). Additionally, two above-plane interactions occur from M72 to F75 and M82 to F77. Site II exhibits a single in-plane S-π interaction from M51 to F56 (Figure 2b). To preserve the hydrophobic core, we choose to make the F37M/F77M double mutant. We similarly choose to produce the F34M/F37M/F77M mutants for TBP6.9. While our mutations were predicted to eliminate a handful of existing S–π interactions, we hypothesized that the chosen Phe-to-Met changes would yield compensatory S–π contacts in return (described below).

### 3.2. dmU1A(F37M/F77M) and TBP6.9(F34M/F37M/F77M) Retain Affinity for hpII and TAR RNAs

**Figure 3.**
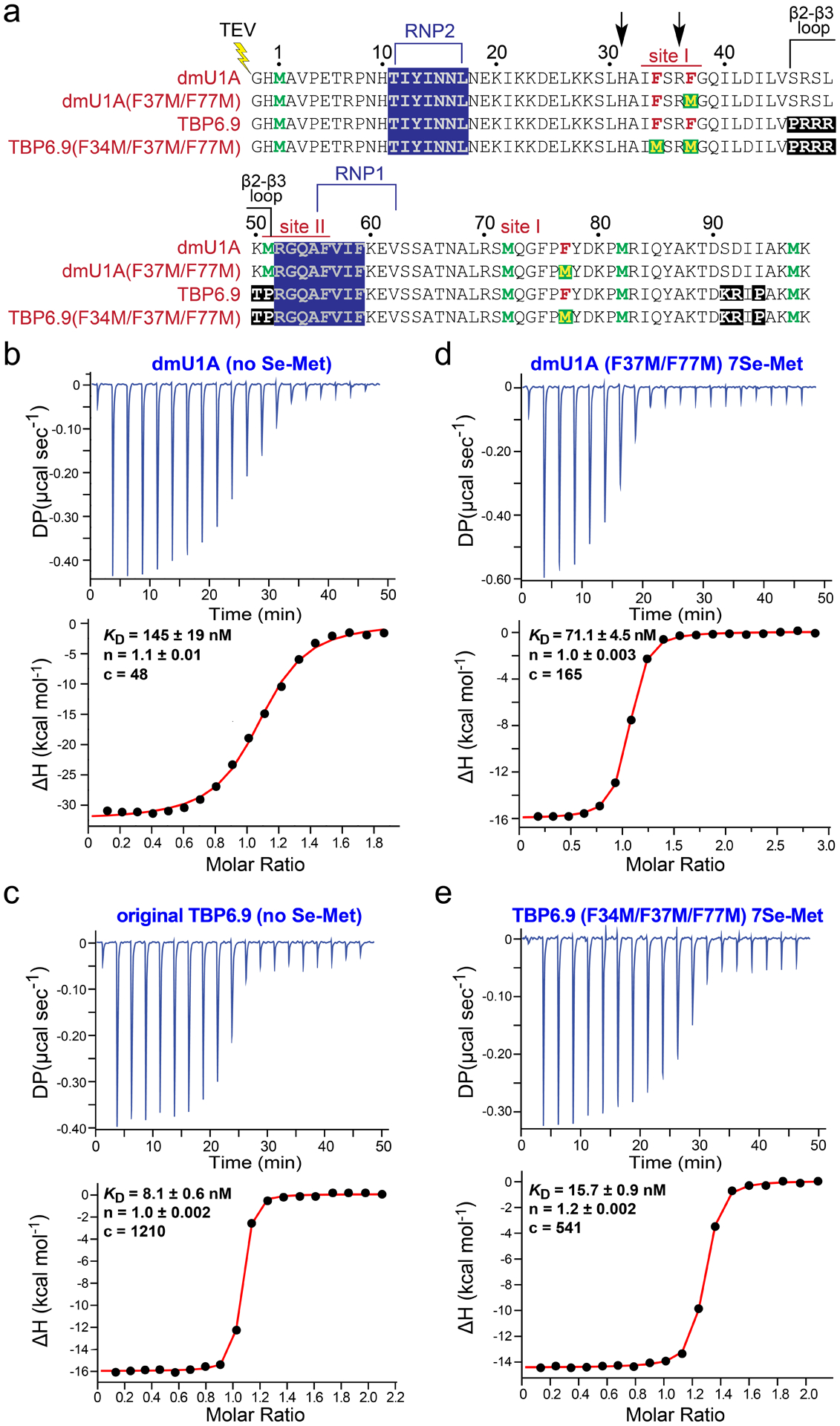
Sequences of dmU1A variants and thermograms of RNA binding to dmU1A(F37M/F77M) and TBP6.9(F34M/F37M/F77M). (**a**) The sequence of human dmU1A crystallized by Nagai [56]; Y31H and Q36R mutants that promote crystallization [57] are depicted by arrows. The conserved RRM binding regions that interact with hpII are labeled RNP2 and RNP1. Met amino acids of parental sequences are green; site I Phe sites described in Figure 2a and those selected for mutagenesis are maroon. Phenylalanine-to-methionine mutations made for this investigation are depicted as green boxes with yellow letters. The lab-evolved β2–β3 loop that promotes HIV TAR RNA binding is labeled. All constructs possess an N-terminal TEV-protease cleavage site (lightning bolt). (**b**) Representative thermogram of dmU1A binding to hpII RNA. Here and elsewhere the apparent *K*_D_, n (ligand to receptor ratio), and c value (i.e., a unitless parameter that describes the curve shape for quality control) are provided for the titration shown; average values are in Table 2. (**c**) Representative thermogram of HIV-1 TAR RNA binding to parental TBP6.9 prepared in the absence of selenomethionine. (**d**) Representative thermogram of hpII RNA dmU1A(F37M/F77M) labeled with selenomethionine. (**e**) Representative thermogram of HIV-1 TAR RNA binding to TBP6.9(F34M/F37M/F77M) labeled with selenomethionine.

We produced the dmU1A(F37M/F77M) and TBP6.9(F34M/F37M/F77M) mutants using synthetic genes of the desired sequence for protein expression in *E. coli*. The proteins were engineered with TEV protease cleavage sites to remove the N-terminal poly-histidine tag after immobilized-metal-affinity chromatography (Figure 3a). Proteins were determined to be of high quality based on Coomassie stained polyacrylamide gels and elution as a single species using size-exclusion chromatography. As an additional quality-control step, we subjected each protein to RNA binding analysis using isothermal titration calorimetry (ITC).

As control experiments, we first measured the equilibrium binding affinity of dmU1A for hpII and TBP6.9 binding to HIV TAR. Both proteins were expressed in bacteria and purified essentially as described [51,52]. U1A has been reported to be a tight binder to hpII RNA based on surface plasmon resonance [86]. Indeed, we measured an average *K*_D_ of 152 ± 7 nM and an average ΔG of −9.15 ± 0.03 kcal mol^−1^ (Figure 3b and Table 2). The average ΔH of −32.0 ± 0.6 kcal mol^−1^ indicates an enthalpy-driven binding process with an unfavorable entropic contribution (average –TΔS of +22.8 ± 0.6 kcal mol^−1^). As for TBP6.9 binding to HIV-1 TAR RNA, we measured an equilibrium binding constant of 8.5 ± 0.4 nM and a ΔG of −10.9 ± 0.1 kcal mol^−1^ (Figure 3c and Table 2). Like dmU1A, TAR binding by TBP6.9 occurs via an enthalpy-driven process with a ΔH of −16.0 ± 0.1 kcal mol^−1^ and an opposing entropic contribution of +5.2 ± 0.01 kcal mol^−1^. This thermodynamic profile is similar to prior measurements of the TBP6.9 interaction with TAR reported by our lab [52].

We next evaluated how the dmU1A(F37M/F77M) and TBP6.9(F34M/F37M/F77M) samples affected binding to their respective target RNAs. We rationalized that the most relevant form of each protein would contain selenomethionine. As such, each analysis was performed on protein samples substituted with selenomethionine. The dmU1A(F37M/F77M) variant yielded a *K*_D_ of 59.7 ± 11.4 nM, which was slightly tighter than dmU1A as indicated by a *K*_rel_ of 0.39 (Figure 3d and Table 2). We hypothesize that selenomethionine enhances the stability of the hydrophobic core of dmU1A(F37M/F77M) to improve RNA binding. Selenomethionine was shown previously to enhance the stability of T4 lysozyme, while leaving the fold unchanged [83]. Indeed, the enthalpic and entropic contributions to hpII binding agreed with trends observed for dmU1A (Table 2).

In contrast, TBP6.9(F34M/F37M/F77M) produced a *K*_D_ of 13.5 ± 2.3 nM, which was 1.6-fold poorer in TAR binding compared to parental TBP6.9 (Figure 3e and Table 2). The triple mutant produced thermodynamic trends comparable to TBP6.9 with a favorable enthalpy (ΔH of −14.5 ± 0.1 kcal mol^−1^) and unfavorable entropy (−TΔS of +3.9 ± 0.1 kcal mol^−1^) (Table 2). The F34M mutation represents a notable variation between dmU1A(F37M/F77M) and the TBP6.9 triple mutant. The F34M mutation was added in the hydrophobic core to produce a TBP6.9 protein with seven methionines for parity with dmU1A(F37M/F77M) (Figure 3a). This was necessary because the lab-evolution and selection processes used to identify TAR binding entailed saturation mutagenesis in the β2–β3 loop [87]. The resulting TBP6.9 sequence did not retain M51, in contrast to the parental U1A protein (Figure 3a). Accordingly, we choose to integrate the F34M variant at site I of the hydrophobic core (Figure 2a) for reasons delineated above. Overall, our binding analyses demonstrated that dmU1A(F37M/F77M) and TBP6.9(F34M/F37M/F77M) are capable of high-affinity binding to their respective RNA targets in the presence of seven selenomethionines.

### 3.3. Evidence that TBP6.9(F34M/F37M/F77M) Targets a TAR Sequence Without the Apical Loop

**Figure 4.**
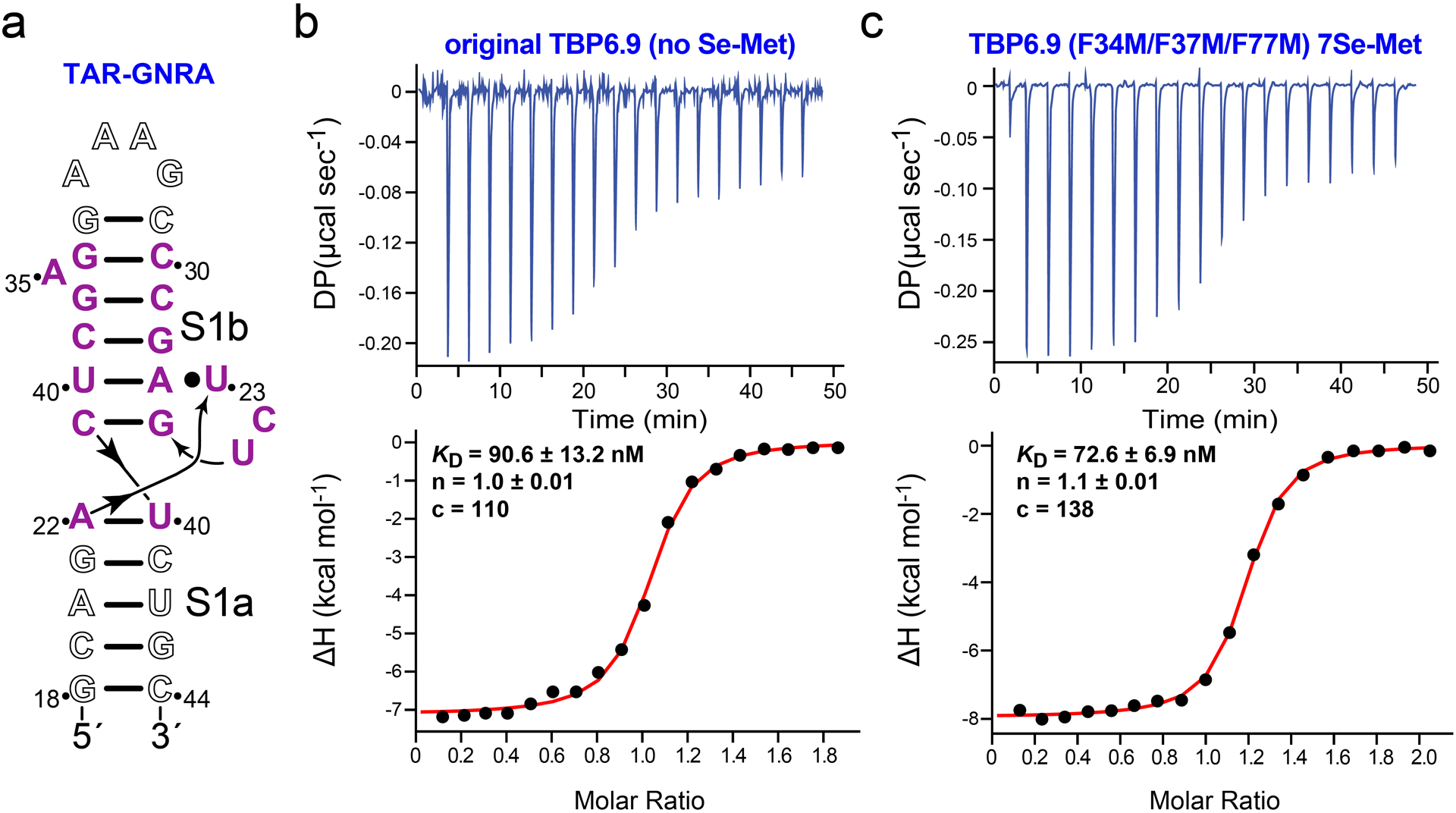
Schematic diagram of the TAR core binding motif for TBP and thermograms of TAR-GNRA binding to TBP6.9 and TBP6.9(F34M/F37M/F77M). (**a**) The TAR internal bulged loop (purple) depicting regions that are non-essential for binding (white) based on the crystal structure and binding experiments [51,52]. The apical loop was substituted with a GAAA tetraloop and a closing C–G pair, which produces optimal thermodynamic stability for this motif [89]. (**b**) Thermogram of GNRA-TAR RNA binding to the parental TAR TBP6.9 prepared without selenomethionine substitution. Here and elsewhere the average thermodynamic parameters are reported in Table 2. (**c**) Thermogram of GNRA-TAR RNA binding to TBP6.9(F34M/F37M/F77M) prepared in the presence of selenomethionine.

To employ TBP6.9(F34M/F37M/F77M) as a crystallization and phasing module, we asked whether the UGG sequence that composes the TAR apical loop could be replaced with a GNRA tetraloop (Figure 4a). Our rationale was that TBP6.9, and related TBPs, appear to recognize the internal bulged loop of TAR in co-crystal structures but not the apical loop, as illustrated in Figure 1b [51,52]. Accordingly, we first evaluated the affinity of parental TBP6.9 for GNRA-TAR. The results revealed a *K*_D_ of 87.8 ± 5.0 nM corresponding to a ΔG of −9.5 ± 0.04 kcal mol^−1^ (Figure 4b and Table 2) Interestingly, the enthalpy of −7.8 ± 0.4 kcal mol^−1^ and entropy of −1.7 ± 0.5 kcal mol^−1^ indicate that both terms contribute favorably to binding (Table 2). This observation pinpoints the TAR apical loop as a region that contributes an unfavorable loss of entropy during TBP6.9 binding to TAR, consistent with the known dynamics of this region in the absence of RNA binding partners (reviewed in [34]). A comparable binding analysis to GNRA-TAR was conducted next for selenomethionine substituted TBP6.9(F34M/F37M/F77M). The results yielded a *K*_D_ of 69.8 ± 2.9 nM, which is nearly indistinguishable from parental TBP6.9 binding to GNRA-TAR (Figure 4c and Table 2); favorable entropic and enthalpic contributions to binding were observed as well. The observation that TBP6.9 recognizes GNRA-TAR with high affinity albeit with 5-fold lower affinity compared to selenomethionine-labeled TBP6.9(F34M/F37M/F77M) binding to TAR is supported by prior experiments conducted on TBP6.7, which differs from TBP6.9 by a Q48R variation in the β2-β3 loop. Specifically, TBP6.7 showed no appreciable loss in TAR affinity based on ELISA analysis when GG in the apical loop was mutated to CC [87]. In contrast, alterations in the closing C30–G34 base pair of the apical loop, or changes at G36, reduced TBP6.7 binding. As such, bases in the apical loop appear dispensable for TBP6.9 binding to TAR. The observed ΔΔG values range from +1.0 to +1.4 kcal mol^−1^ when comparing TBP6.9 binding to TAR versus GNRA-TAR (Table 2), which is energetically equivalent to two hydrogen bonds or a S–π interaction [51,84]. Although we have not yet proven that TBP6.9(F34M/F37M/F77M) is efficacious as a means to promote crystallization and phasing, our ITC results lead us to speculate that the internal bulged loop of TAR (Figure 1b) can be transplanted into duplex regions capped by essential helical or loop sequences (e.g., loop 4 of the preQ_1_-III riboswitch, which forms an H-type pseudoknot [88]) to facilitate crystallographic analysis.

### 3.4. Crystallization of dmU1A(F37M/F77M) Alone and in Complex with RNA

**Figure 5.**
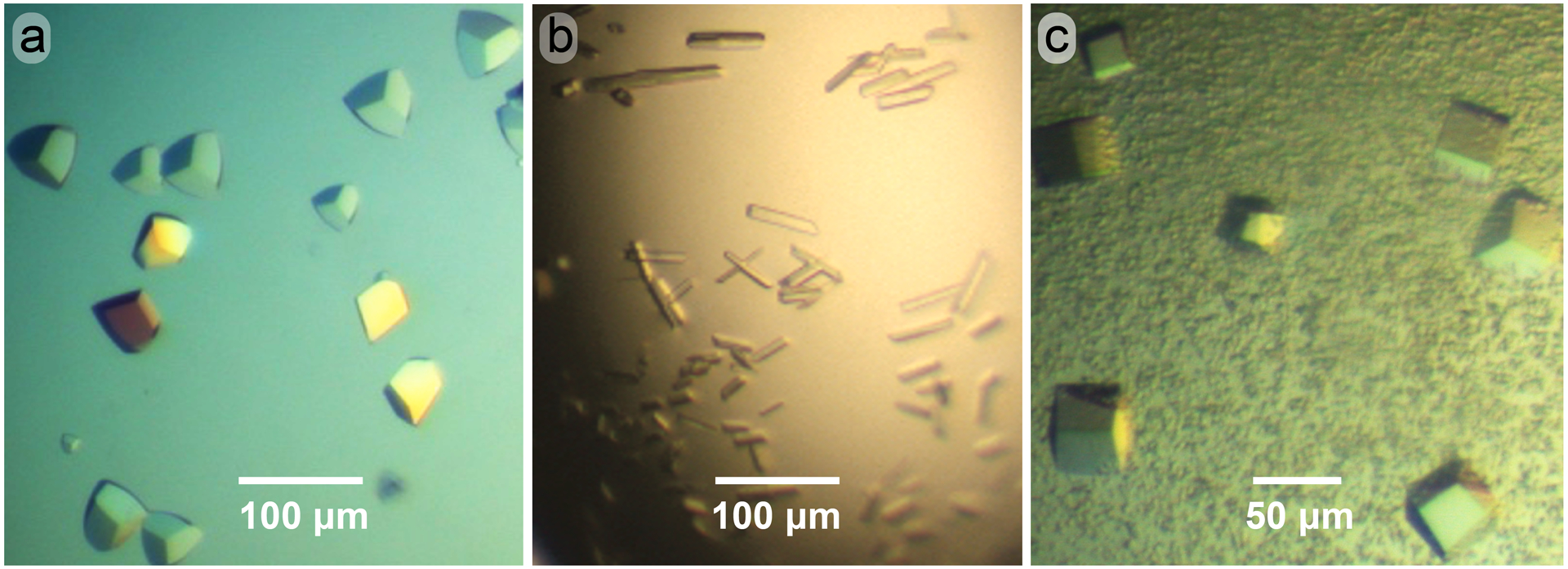
Representative crystals of dmU1A(F37M/F77M) labeled with selenomethionine. (**a**) Trigonal crystals grown in the absence of RNA from solutions of Na-acetate buffered at neutral pH. Here and elsewhere, crystals were photographed under polarized light. (**b**) Rods of dmU1A(F37M/F77M) bound to the hpII 21-mer RNA. Crystals grew from solutions of polyethylene glycol buffered at neutral pH. (**c**) Cubic crystals of dmU1A(F37M/F77M) in complex with a preQ_1_-II riboswitch 84-mer. Crystals grew from lithium sulfate buffered at pH 8.5.

We next addressed whether the dmU1A(F37M/F77M) variant was amenable to crystallization. U1A containing various mutations has been crystallized alone [57,90,91], with hpII RNA [56,57] and in complex with various target RNAs modified to contain the hpII loop (Figure 1a) in place of functionally dispensable loop sequences (Table 1). Based on a prior report of dmU1A crystallization from malonate in the absence of RNA, and experience within the community using acetate as a crystallization agent [92,93], we identified a trigonal crystal form of the selenomethionine-substituted dmU1A(F37M/F77M) variant grown from 2.7–3.1 M Na-acetate solutions buffered in a pH range from 6.5–7.0 (Figure 5a). Crystals reached a maximum dimension of 60 μm in 1 week at 20 °C, appeared free from growth defects and showed orientation-dependent birefringence. Although acetate at high concentration can serve directly as a cryoprotectant like other organic acid salts such as malonate or Tacsimate^™^ [89] we supplemented the 3.0 M Na-acetate mother liquor with glycerol to produce a 5% (*v/v*) solution prior to flash freezing. In this manner, crystals were preserved for x-ray diffraction analysis (described below).

Our next goal was to crystallize dmU1A(F37M/F77M) in complex with the hpII RNA 21-mer (Figure 1a), as described by Nagai and co-workers for dmU1A and various hpII constructs [57]. As reported for the dmU1A-hpII 21-mer complex, we observed crystals under numerous conditions including some with growth deformations, ‘scissor’ morphologies, and polycrystallinity [57]. These were attained from a variety of conditions including high (NH_4_)_2_SO_4_, Tris-HCl pH 7.0, and spermine-HCl, as reported [57]. However, our best single crystals grew from solutions of polyethylene glycol (PEG) 4000 buffered by MOPS pH 7.0, and low concentrations of NaCl. Crystals also grew from PEG6000 buffered at pH 7.5 by Tris-HCl. The latter crystals formed elongated parallelepipeds that appeared in ~1 week at 20 °C and were of sufficient size for x-ray diffraction experiments (Figure 5b). Unfortunately, this crystal habit showed no detectable diffraction on our in-house source. Nagai and co-workers similarly noted that co-crystals of the dmU1A-hpII complex prepared from solutions of PEG “exhibited no diffraction, so this precipitant was rarely used in screens” [57]. Although this is a negative result, it suggests that dmU1A(F37M/F77M) behaves similarly to parental dmU1A and that the crystals shown in Figure 5b are not salt, which would likely show very strong Bragg reflections in a range between ~10 to 1 Å resolution [80].

We also grew cubic crystals of selenomethionine-substituted dmU1A(F37M/F77M) in complex with a *Lactobacillus rhamnosus* preQ_1_-II riboswitch 84-mer from high concentrations of lithium sulfate (Figure 5c), based on an in-house crystallization screen [66]. The hpII stemloop was substituted into the non-conserved P1 hairpin of the wildtype riboswitch [63] to allow dmU1A(F37M/F77M) binding. Crystals grew in ~1 week at 20 °C and lacked detectable birefringence, consistent with a high-symmetry space group. Indeed, x-ray diffraction analysis showed that crystals belong to cubic space group *F*23 with cell dimensions *a* = *b* = *c* = 240.2 A° with α = β = γ = 90°. The estimated solvent content is 55–70% [94], suggesting 2 or 3 complexes per asymmetric unit. Crystals showed diffraction to 4.5 Å resolution, but the data were complete to only 5.5 Å resolution (Supplementary Table S1). The overall *R*_sym_ was 10.7% (54.3%) with an *I*/σ(*I*) of 10.7 (1.7), where parenthetical values indicate the highest resolution shells. Although the poor resolution could be attributable to the small crystal size of 50 μm *×* 50 μm *×* 20 μm, it is also likely that the crystallization construct requires further optimization. Indeed, methods that employ the dmU1A protein for co-crystallization with RNAs containing hpII call for optimization steps that include varying the length of the hpII helix in single base pair increments to improve x-ray diffraction [53–55]. This process can be labor and resource intensive, and we did not undertake such optimization efforts here. Nonetheless, our results demonstrate that the dmU1A(F37M/F77M) variant labeled with selenomethionine crystallizes in the absence and presence of RNA.

### 3.5. Structure Determination and Quality-control Analysis of the dmU1A(F37M/F77M) Variant

**Figure 6.**
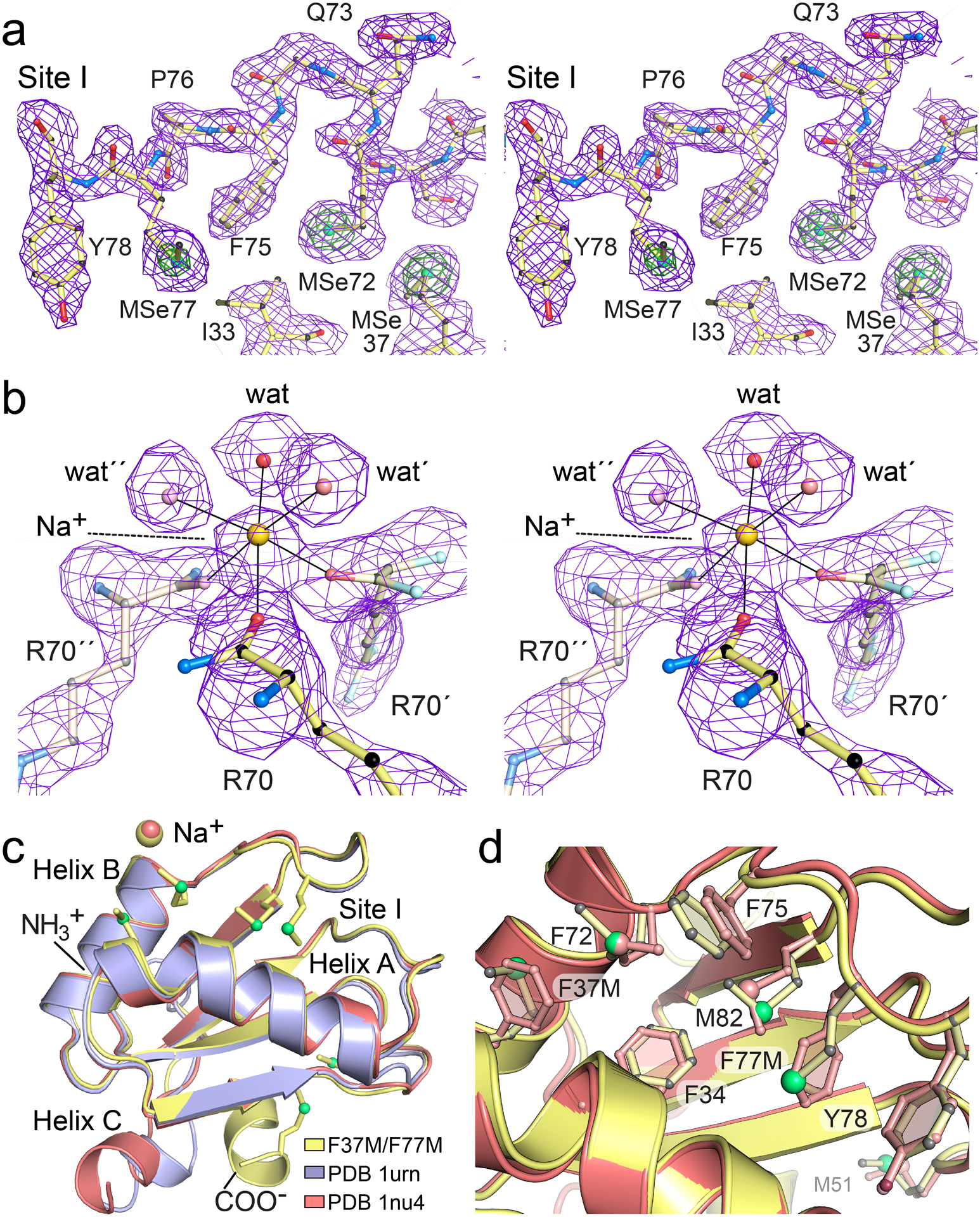
Representative electron density maps of the selenomethionine-labeled dmU1A(F37M/F77M) variant and comparison of dmU1A structures. (**a**) Stereo view of 2m*F*_o_−D*F*_c_ electron density (purple) contoured at 1.0 σ (purple) and 5.0 σ (green) for the site I hydrophobic pocket. Amino acids including F37M and F77M are depicted as ball-and-stick models. (**b**) Stereo view of a simulated-annealing composite omit map depicting a sodium ion on the crystallographic 3-fold axis. The ion is coordinated by three carbonyl oxygens from R70 and three waters (labeled ‘wat’). For clarity, only the R70 amino acid, the Na^+^ ion and an inner sphere water were omitted from the phase calculation; the amide nitrogen of S71 is shown to indicate the peptide bond but was included in the phase calculation. (**c**) Pairwise superpositions of dmU1A (salmon) prepared from sodium malonate [90] and dmU1A from the protein-RNA complex (slate) [56] with the dmU1A(F37M/F77M) mutant of this investigation (yellow) prepared from Na-acetate and labeled with selenomethionine. (**d**) Close-up view of site I from the superimposed dmU1A and dmU1(F37M/F77M) mutant from panel **c**.

We next sought to understand how the F37M and F77M substitutions influenced the structure of the dmU1A hydrophobic core. Accordingly, we collected X-ray diffraction data on flash cooled crystals depicted in Figure 5a and determined the structure by molecular replacement using the dmU1A component of the dmU1A-hpII complex [56]. X-ray diffraction data were collected to 2.20 Å resolution. The data set was complete and showed a high signal-to-noise ratio of 61.2 (20.2 in the highest resolution shell) with 10-fold redundancy (Table 3). The quality of the data was indicated further by an *R*_merge_ of 2.9% (7.6% in the highest resolution shell) and a correlation coefficient between half data sets (CC_1/2_) of 0.995 in the highest resolution shell. The dmU1A(F37M/F77M) structure was refined to an overall *R*_cryst_ of 16.8%, with a 4.7% difference between *R*_work_ and *R*_free_, signifying the model is not overfit [99]. Representative electron density reveals a well-ordered core at the site of the F37M and F77M mutations (Figure 6a). All amino acids are positioned in the most-favored regions of the Ramachandran plot and the overall average *B*-factor was 20.2 Å^2^. Although some electron density was visible for the first methionine, the polypeptide mainchain was not appreciably visible until V3.

An ordered sodium was a notable feature of electron density maps as well (Figure 6b). This cation is present at 3.0 M levels in the crystallization medium and mediates crystal contacts about the proper threefold axis. The ion receives octahedral coordination from the crystallographically-related carbonyl oxygens of R70, R70´, and R70´´, as well as three symmetry-related water molecules. The use of ligand groups without formal charges is consistent with the single positive charge carried by this ion [100]. Oxygen-to-sodium distances are 2.4 to 2.5 Å, in accord with prior accounts of alkali metal interactions in proteins [100,101]. The sodium ion is also positioned at the carboxylic end of helix B where it could benefit from the local helix dipole [102]. The observed details of the Na^+^ ion coordination sphere are a further indicator of quality for the structural model.

### 3.6. Comparison of the dmU1A(F37M/F77M) Variant to a dmU1A Structure Prepared from Malonate

The preparation of our dmU1A(F37M/F77M) crystals from sodium acetate was reminiscent of a prior study of dmU1A wherein crystals were grown from sodium malonate [90]. To identify similarities and differences in these structures each prepared from organic acid salt we performed an all-atom superposition of these molecules (Figure 6c, yellow versus salmon-colored molecules). The average *xyz* displacement was 2.07 Å with the greatest difference observed at helix C, which moves closer to helix B and farther away from the RNA binding surface in the malonate structure. Removal of helix C from the comparison reduced the average displacement to 0.94 Å (i.e., Δ88–98). We then compared the helix C conformation in our Na-acetate structure to that of the dmU1A-hpII complex [56]. We found that the position of helix C in the dmU1A(F37M/F77M) variant is oriented nearly perpendicularly compared to that of the dmU1A-hpII complex (average displacement of 1.78 Å for all atoms and 0.882 Å when helix C is removed). The helix appears to move as a rigid body to accommodate RNA binding (Figure 6c, yellow versus purple structures), which is necessary to avert a clash between hpII and the helix C conformation observed in our Na-acetate structure. A take-home message is that organic acid salts do not promote a specific helix C conformation and that the C-terminal region is malleable for RNA recognition, as demonstrated by mainchain displacements as great as 10 Å. Interestingly, the dmU1A-derived TBP6.9 protein does not use its C-terminus to recognize HIV TAR RNA (Figure 1b). In fact, this region of the polypeptide chain was disordered in all TBP–TAR complexes [51,59]. The average all-atom displacement of TBP6.9 superimposed on the dmU1A(F37M/F77M) variant was 1.33 Å for residues 5–91. The greatest conformational difference was localized in the lab-evolved β2–β3 loop that recognizes TAR RNA.

Comparison of the dmU1A(F37M/F77M) variant to the dmU1A structure prepared from Na-malonate revealed that each structure coordinates a cation near R70 of helix B (Figure 6c). Remarkably, both structures employ the same R70 carbonyl oxygens and comparable waters for octahedral ion coordination (e.g., Figure 6b), which is centered on a crystallographic 3-fold axis present in each different space groups. Although a Na^+^ ion was assigned to the dmU1A(F37M/F77M) variant grown from 3.0 M Na-acetate, Mg^2+^ was modeled for the dmU1A structure prepared from 2.2 M Na-malonate. Both structures show similar ion-to-oxygen coordination distances of 2.46 ± 0.10 for dmU1A and 2.50 ± 0.05 Å for dmU1A(F37M/F77M). Given the identical number of electrons for Mg^2+^ and Na^+^, crystallization from 2.2 M Na-malonate, and the observation of slightly longer ion-to-oxygen coordination distances for Na^+^ (2.53 ± 0.17 Å) [103] compared to Mg^2+^ (2.06 ± 0.02 Å) [104,105], it appears that R70 is a nexus for Na^+^ coordination in the dmU1A structure. It has been noted that Na^+^ is frequently mistaken for Mg^2+^ in crystal structures, and the greater formal charge of alkaline earth metals is frequently associated with coordination by one or more acidic ligands [100]. In contrast, no evidence was observed for comparable cation coordination in complexes of dmU1A-hpII [56] or TBP6.9-TAR [52,59]. Nonetheless, we posit that inclusion of Na^+^ ions could prove helpful to promote crystallization of dmU1A–RNA complexes.

### 3.7. Site I of dmU1A(F37M/F77M) Maintains the Core Fold but Makes new S–π Interactions

We next asked how the F37M and F77M mutations were accommodated in the site I hydrophobic core of U1A. We compared our double mutant to the dmU1A structure grown from Na-malonate. Interestingly, F37M and F77M seem well accommodated by environments that evolved to contain aromatic groups (Figure 6d). The selenomethionine side chain at position 37 appears coplanar with the corresponding phenylalanine aromatic ring of dmU1A with only the ε-methyl group of the former residue breaking planarity. Nearby M72 appeared to adjust in response to the smaller girth of the F37M change by rotating its ε-methyl group by ~180° about its χ^3^ dihedral angle. This variation allows a closer positioning of the F75 aromatic ring, which appears to form a new, above-plane Se-π contact with F37M. M82 similarly rotates its ε-methyl group by ~180° to maintain a S–π contact with F34. F77M reveals another way to accommodate the selenomethionine sidechain within the volume of an aromatic ring. In this instance, the sidechain atoms do not superpose onto those of phenylalanine but the Seδ and Cε atoms adopt a coplanar orientation relative to the dmU1A aromatic ring. This conformation allows F77M to engage in a new Se-π contact with the edge of Y78. The results demonstrate the remarkable adaptability of the U1A core and its ability to make numerous small compensatory interactions. These observations provide confidence that the TBP6.9(F34M/F37M/F77M) triple mutant will be accommodated similarly.

### 3.8. Substructure Determination and SAD Phasing of the dmU1A(F37M/F77M) Variant

**Figure 7.**
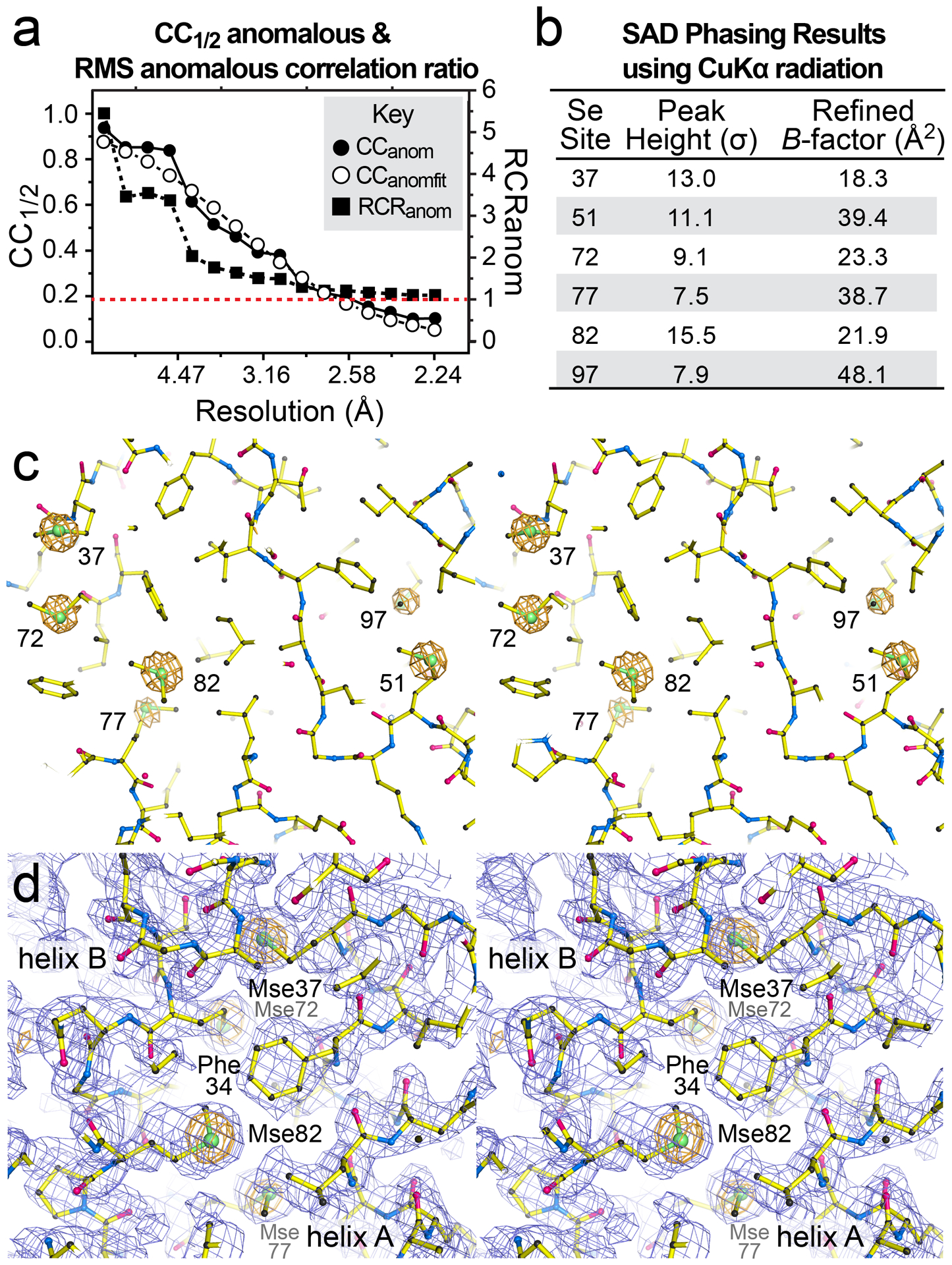
Selenomethionine anomalous signal, substructure determination and representative noise-filtered SAD-phasing electron-density map of dmU1A(F37M/F77M). (**a**) (*Left ordinate*) Plot of the anomalous difference correlation coefficients CC_anom_ and CC_anomfit_ as a function of resolution. (*Right ordinate*) Plot of RMS correlation ratio for anomalous diffraction (RCR_anom_) as a function of resolution. A dashed red line indicates a value of 1.0 for the RCR_anom_ signal. Parameters are defined as described in Aimless [86]. (**b**) Results of Se-substructure identification using a hybrid search [76] and values of the refined *B*-factors from the corresponding model in Figure 6. The heights of the six selenium anomalous difference Fourier peaks were derived from Phaser EP [72]. (**c**) Stereo view of the anomalous difference Fourier peaks from panel *b*, shown in the correct hand and contoured at 5σ; the final refined model of dmU1A(F37M/F77M) from Figure 6 is shown. (**d**) The noise-filtered SAD phasing map contoured at 1.0σ (slate blue) shows recognizable secondary structure features. The refined model from Figure 6 is shown and fits well to the experimental electron density. The anomalous difference Fourier map contoured at 4σ is shown to emphasize the locations of the site I Se atoms.

To be useful as a phasing tool, we next assessed the ability to detect an anomalous signal from the selenomethionine-labeled dmU1A(F37M/F77M) variant. Our in-house data-collection strategy was designed to record the anomalous diffraction data for the Se atoms (*f*´´ of 1.27 e^−^ at 1.5418 Å, which is 2.3-fold higher than *f*´´ for sulfur at 0.56 e^−^). As an indicator of the resulting anomalous signal quality, we plotted the CC_anom_ and CC_anomfit_ correlation coefficients for the diffraction data (Figure 7a). A CC_anom_ ≥ 0.15 is evidence of an anomalous signal. The resolution limit of the useful anomalous signal was estimated based on a linear fit [97], which suggested a cutoff of ~2.58 Å resolution. In contrast, the RCR_anom_ metric indicates a significant anomalous signal when the fit value is >1 [86]. Our analysis suggests a cutoff of ~2.20 Å resolution (Figure 7a), where the RCR_anom_ signal is 1.11 in the highest resolution shell (Table 3).

We next asked whether the Se substructure could be located using a hybrid substructure search as implemented in Phenix [73,76,106]. Indeed, six of the seven Se atoms were located, including those resulting from the F37M and F77M mutations (Figure 7b,c). Sites were input into Phaser-EP [72] for refinement and SAD phasing in both substructure hands. The resulting anomalous difference peaks for Se ranged from 7.5σ to 15.5σ; the overall figure of merit was 0.43 and the log-likelihood gain was 329 to 2.20 Å resolution. When the new Se sites from the F37M and F77M mutations were excluded from the phase calculation, the resulting figure of merit and log likelihood gain values were diminished to 0.35 and 141 to 2.20 Å resolution. Although the Se resulting from the F77M mutation produced the lowest anomalous difference peak height, its signal was still comparable to that of Mse97. In contrast, the Se resulting from the F37M mutation produced the second highest anomalous signal after that from Mse82 (Figure 7b,c). Electron density maps calculated using phases from each hand of the Se substructure were subjected next to noise filtering [73,77]. Visual inspection of electron-density maps revealed the correct hand based on clearly recognizable secondary-structure features that readily accommodated the refined dmU1A(F37M/F77M) model (Figure 7d). The experimental SAD phases were used for autobuilding [75], which produced a model with *R*_work_ and *R*_free_ values of 26 and 31%, respectively.

In the model resulting from MR, the six Se atoms refined to an average *B*-factor of 27.4 Å^2^, including Mse97 at the C-terminus, which is often disordered (i.e., in cases when only three Se atoms were reported in Table 1). The Se *B*-factors ranged from 18.3 Å^2^ for the F37M variant to 48.1 Å^2^ for Mse97 (Figure 7b). The Se at site F77M yielded a B-factor of 38.7 Å^2^; this value is comparable to that from Mse51 in site II, which refined to 39.4 Å^2^. Overall, this analysis demonstrates that the added selenomethionines are ordered, provide detectable anomalous diffraction signals, and that these sites were useful for phasing dmU1A(F37M/F77M). This variant should prove useful to improve the measurable anomalous and dispersive diffraction signals required for SAD and MAD phasing of dmU1A(F37M/F77M)-RNA complexes.

## 4. Conclusions

Structured RNA molecules can be difficult to crystallize in a form that yields high-resolution diffraction, making it challenging to accurately measure anomalous or dispersive intensity differences required for de novo SAD and MAD phasing. Indeed, numerous instances have been reported in which dmU1A was successful in promoting crystallization but additional experimental phasing approaches were needed beyond MAD or SAD including: Ir(NH_3_)_6_(III)-based phasing [42,48], MR and MAD [46], or MAD with phase combination from other heavy-atom derivatives [40]. A survey of dmU1A-RNA complexes determined by MAD or SAD phasing revealed that only three or four selenomethionines are ordered typically in the protein-RNA complex (Table 1). Assuming an Δ*f´´*_max_ of 4e^−^ and an interwavelength Δ*f´* of −7e^−^ [107,108], we calculated that the anomalous and dispersive signals from a theoretical dmU1A-RNA 50-mer complex would yield Bijvoet and dispersive differences of 2.3–2.7% and 2.0–2.3% [62], based on the diffraction ratios from 3–4 Se sites. These intensity differences are close to the detectable signal limit for most RNA data sets based on *R*_sym_ metrics. Addition of two ordered selenium atoms to the dmU1A crystallization module as shown here could enhance the Bijvoet and dispersive differences for a hypothetical U1A-RNA 50-mer complex by as much as 20 to 30% [62]. The dmU1A(F37M/F77M) variant could also facilitate phasing of larger U1A-RNA complexes.

We further demonstrated that a dmU1A variant containing the F37M/F77M double mutant retained binding to hpII RNA and could be crystallized in the presence and absence of RNA. The resulting crystal structure indicated that the F37M and F77M mutations were well packed in the hydrophobic core and produced compensatory Se-π interactions that were accompanied by modest sidechain adjustments to nearby hydrophobic residues. In total, six ordered selenium atoms were observed in electron density maps, and each produced a measurable anomalous difference signal. The useful limit of the anomalous signal was between 2.58 to 2.20 Å based on CC_anomfit_ and RCR_anom_ calculations (Table 3), as well as Se SAD phasing from the anomalous differences (Figure 7). Based on the core packing of the dmU1A(F37M/F77M) variant, it is conceivable that additional methionines could be added to augment the selenium diffraction ratio. Indeed, the F34M mutation was included in the TBP6.9(F34M/F37M/F77M) triple mutant to provide six ordered selenium atoms for phasing in the context of the TAR RNA internal bulged loop motif. It is conceivable that sufficiently close Se atoms resulting from core mutations at site I would appear as super-selenium when analyzed at low resolution. This concept is similar to disulfides used in SAD phasing, which appear as single super-sulfurs in anomalous difference Patterson maps [109]; albeit the closest selenium atoms in the dmU1A(F37M/F77M) structure are a distant 5.0 Å apart. Nevertheless, the presence of six ordered selenium atoms is expected to improve the phasing capacity of the already successful dmU1A crystallization and phasing module. Although untested, we posit that the TBP6.9(F34M/F37M/F77M) variant will prove useful for experimental MAD and SAD phasing of structures judiciously engineered to contain the TAR internal bulged loop within non-conserved duplex regions of the target RNA sequence.

## Supplementary Material

Supplementary Material

## Figures and Tables

**Table 1.** Representative Structures in Complex with dmU1A or TBP.

Sample Name	PDB ID	Phasing Method	No. Se Sites	Ref.
SAM-VI riboswitch	6las	SAD^a^	3–4	[36]
NAD^+^-I riboswitch	7d7v	SAD	3 of 4	[37]
HDV ribozyme	1sjf	MAD^b^	4 of 4	[38]
HDV ribozyme	1drz	MAD	4 of 4	[39]
group I intron	1u6b	MAD^c^	4 of 4	[40]
glutamine riboswitch	5ddp	MR^d^	n/a	[41]
glycine riboswitch	3p49	MAD^e^	n/a	[42]
cyclic-di-AMP riboswitch *ydaO*	4w90	MAD	3 of 4	[43]
c-di-GMP riboswitch	3iwn	MR^d^	n/a	[44]
c-di-GMP riboswitch	3irw	MAD^e^	n/a	[45]
aminoacyl tRNA synthetase ribozyme	3cun	MAD/MR^c^	3 of 4	[46]
tetracycline artificial riboswitch	3egz	MAD	3 of 4	[47]
class I ligase ribozyme	3hhn	MAD^e^	n/a	[48]
pre-cleaved *glmS* ribozyme	3g8S	MR^d^	n/a	[49]
K-turn-L7Ae complex	4c4w	MR^d^	n/a	[50]
TAR RNA in complex with TAR binding protein 6.7	6cmn	MR^f^	n/a	[51]
TAR RNA in complex with TAR binding protein 6.9	6xh0	MR^g^	n/a	[52]

a Single-wavelength anomalous diffraction.

b Multiwavelength anomalous diffraction.

c Additional phasing approaches were required beyond the use of the Se-Met atoms in U1A.

d Molecular replacement (MR) using the dmU1A complex with the hpII loop.

e MAD phasing was used but Ir(NH_3_)_6_ was the basis for phasing.

f The search model was dmU1A alone.

g The search model was the TAR-TBP6.7 complex.

**Table 2.** Isothermal titration calorimetry of wildtype and mutant dmU1A and TBP6.9.

Sample	K_D_ nM	n	ΔH kcal mol^−1^	−TΔS kcal mol^−1^	ΔG kcal mol^−1^	*K*_*rel*_^a^
dmU1A^b^	152.0 ± 7.0^c^	1.0 ± 0.02	−32.0 ± 0.6	22.8 ± 0.6	−9.2 ± 0.03	N/A
dmU1A 7Se-Met (F37M/F77M)^b^	59.7 ± 11.4	0.95 ± 0.05	−19.1 ± 3.0	9.4 ± 2.9	−9.7 ± 0.12	0.39
TBP6.9 (WT)^d^	8.5 ± 0.4	1.0 ± 0.02	−16.0 ± 0.1	5.2 ± 0.01	−10.9 ± 0.1	N/A
TBP6.9 7Se-Met (F34M/F37M/F77M)^d^	13.5 ± 2.3	1.2 ± 0.02	−14.5 ± 0.1	3.9 ± 0.1	−10.6 ± 0.1	1.6
TBP6.9 *(WT)*^e^	87.8 ± 5.0	0.94 ± 0.04	−7.8 ± 0.4	−1.7 ± 0.5	−9.5 ± 0.04	10.3
TBP6.9 7Se-Met (F34M/F37M/F77M)^e^	69.8 ± 2.9	1.1 ± 0.01	−7.2 ± 0.8	−2.4 ± 0.9	−9.6 ± 0.03	8.2

a The ratio of *K*_D_ values of the mutant divided by the matched wildtype (i.e., dmU1A or TBP6.9).

b The RNA sample in the cell was the hpII 24-mer in Figure 1a.

c Errors represent standard errors of the mean for multiple measurements.

d The RNA sample in the cell was the TAR RNA 27-mer in Figure 1b.

e The RNA sample in the cell was the GNRA-TAR RNA 30-mer depicted in Figure 4a.

**Table 3.** Diffraction and refinement statistics of dmU1A(F37M/F77M) Se-Met.

Data Collection	
Wavelength (Å)	1.5418
Resolution range (Å)	27.88–2.20 (2.27–2.20)
Space group	*P* 3 2 1
*a = b, c* (Å)	64.4, 46.8
α = β, γ (°)	90.0, 120
Unique reflections	10,961
Multiplicity	10.0 (4.8)
Completeness (%)	100.0 (100.0)
Mean *I*/σ(*I*)	61.2 (20.2)
*R*_merge_ (%)^b^	2.9 (7.6)
*R*_p.i.m._ (%)^c^	0.9 (3.8)
CC_1/2_ (%)^d^	1.00 (0.995)
CC_anom_ (%)^e^	0.46 (0.10)
RCR_anom_^f^	1.65 (1.11)
**Refinement**	
No. reflections (all/work/test)	11519/10,961/558
*R*_*cryst*_/*R*_work_/*R*_free_ (%)^g^	16.8/16.6/21.3
No. of atoms:	
protein	776
waters	85
Na/Acetate/βME	13
Se	6
R.M.S. deviations:	
bonds (Å)	0.003
angles (°)	0.53
ML coordinate error (Å)	0.19
Clashscore^h^	0.0
Molprobity score^h^	0.50
Ramachandran Plot (%):	
most favored	100
outliers (%)	0
*B*-factors overall (Å^2^):	20.2
protein	19.6
waters	24.3
Na/Acetate/βME	30.8
Se atoms	27.4

a Statistics for the highest-resolution shell are shown in parentheses.

b *R*_merge_ = ∑_hkl_∑_i_|*I*_i_ − <*I*>|/∑_hkl_∑_i_ |*I*_i_| where *I*_i_ is an intensity *I* for the *i*^th^ measuremen of a reflection with indices *hkl* and <*I*> is the weighted mean of all measurements of *I*.

c *R*_p.i.m_. = ∑_hkl_ (1/(n−1))∑_i_|*I*_i_ − <*I*>|/∑_hkl_∑_i_ |*I*_i_| where n is the number of observations of intensity *I*_i_ [95].

d CC_1/2_, correlation coefficient between intensities of random half-dataset [96].

e CC between anomalous pairs measured in the intensity data, as defined in Aimless [97].

f RMS Correlation Ratio (RCR) defined in Aimless [97].

g *R*_cryst_ = Σ_hkl_||*F*_obs_(*hkl*)|−|*F*_calc_(*hkl*)||)/Σ_hkl_|*F*_obs_(*hkl*)| for all reflections, *R*_work_ is for ~95% of reflections, and *R*_free_ is for ~5% of the reflections chosen randomly and excluded from refinement.

h Calculated using the program Molprobity [98].
