# Supplementary material for: Affinity and Structural Analysis of the U1A RNA Recognition Motif with Engineered Methionines to Improve Experimental Phasing
Source: Crystals (Basel). Author manuscript; Available in PMC 2021 Mar 26. (PMC7996396; doi:10.3390/cryst11030273)
Supplement: Supplementary Material [file NIHMS1682169-supplement-Supplementary_Material.docx]

Supplementary Information

**Affinity and structural analysis of the U1A RNA recognition motif with engineered methionines to improve experimental phasing**

Yoshita Srivastava^1,‡^, Rachel Bonn-Breach^1,‡^ , Sai Shashank Chavali^1,‡^, Geoffrey M. Lippa^1,3^, Jermaine L. Jenkins^1,2^ and Joseph E. Wedekind^1,*^

^1^Department of Biochemistry & Biophysics and Center for RNA Biology, University of Rochester School of Medicine & Dentistry, Rochester NY 14642, USA

^2^Structural Biology and Biophysics Facility, University of Rochester School of Medicine & Dentistry, Rochester NY 14642, USA

^3^Present address: Division of Biology, Alfred University, Alfred, NY 14802

*Correspondence: joseph.wedekind@rochester.edu; Tel.: +1 585 273-4516

**Supplementary Methods**

*Equations for ITC fitting*

ITC data for Figures 3 and 3 were fit to a 1:1 binding model. The variables are defined as follows:

*K* = Binding constant

*n* = number of sites on the receptor

V_o_ = Active cell volume

*M*_t_ = Bulk concentration of macromolecule in V_o_

*M* = Free concentration of macromolecule in V_o_

*X*_t_ = Bulk concentration of ligand

*X* = Free concentration of ligand

*θ* = Fraction of sites occupied by ligand X

(eqn. 1)

$$K=\frac{\theta}{\left( 1-\theta\right) [X]}$$

(eqn. 2)

$$X_{t}=\left[ X \right]+n\theta M_{t}$$

Combining equations (1) and (2)

(eqn. 3)

$$\theta^{2}-\theta\left[ 1+\frac{X_{t}}{nM_{t}}+\frac{1}{nKM_{t}} \right]+\frac{X_{t}}{nM_{t}}=0$$

The heat content (Q) of the solution is considered relative to unliganded species contained in the active cell volume V_o_ at fractional saturation θ is given by:

(eqn. 4)

$$Q=n\theta M_{t}\Delta HV_{o}$$

Here, ΔH denotes the molar heat of ligand binding; *θ* is solved from quadratic eqn. 3, which when substituted into eqn. 4 gives the total heat content Q. Combining equations gives:

(eqn. 5)

$$Q=\frac{nM_{t}\Delta HV_{o}}{2} [1+\frac{X_{t}}{nM_{t}}+\frac{1}{nKM_{t}}-\sqrt{\left( 1+\frac{X_{t}}{nM_{t}}+\frac{1}{nKM_{t}} \right)^{2}-\frac{4X_{t}}{nM_{t}}}$$

The expression in eqn. 5 is suitable for any given n, *K* and ΔH at the end of every *i*^th^ injection. However, eqn. 5 can be used only to calculate total heat content of solution contained in the volume V_o_. Since every injection releases an additional volume of the ligand (ΔV_i_ = injection volume), the change in heat content upon injection (i.e., *i-1* to *i*^th^) is the parameter of interest. Accordingly, for each injection a correction is made for the additional added volume; this assumes mixing and associated kinetics are fast. Hence, the heat effect resulting from the liquid in the displaced volume is equivalent (~50%) to the heat effect due to the remaining volume V_o_. The expression for heat released after every *i*^th^ injection ($\Delta Q\left( i \right)$) is given by:

(eqn. 6)

$$\Delta Q\left( i \right)=Q\left( i \right)+\frac{dVi}{V_{o}} \left[ \frac{Q\left( i \right)+Q\left( i-1 \right)}{2} \right]-Q(i-1)$$

The process of fitting ITC data subsequently involves four steps: *i*) preliminary estimations of n, *K*, and Δ H — are made by the ITC software; *ii*) the ΔQ(i) is then calculated for each injection and the values are compared with the heats measured from experimental injections; *iii*) this improves the initial estimates of n, *K*, and ΔH by standard Marquardt fitting methods; and *iv*) the procedure is iterated until there is no substantial improvement of the fit. Note; this description was adapated from reference [1].

**Figure S1. Sephacryl S-300 HR (16/60) size-exclusion chromatography profiles for dmU1A and dmU1A(F37M/F77M) and molecular-weight standard curve**. (***a***) A representative gel filtration profile is shown for dmU1A prepared in the absence of selenomethionine. The observed elution volume (V_e_) was 97.9 mL. The protein concentration was ~150 μM; see Section 2.2 of main text for details. (***b***) Gel filtration profile for dmU1A(F37M/F77M) labeled with selenomethionine. The sample concentration was ~150 μM The observed total column volume, V_t_, was 102.0 mL. (***c***) Gel filtration profiles were measured for MW standards aldolase A, BSA, ovalbumin and chymotrypsinogen A. Blue dextran was used to define the void volumne (V_o_) of 39.14 mL. The bed volume defined by the manufacturer was 120.36 mL. (***d***) A molecular weight calibration curve was generated by plotting K_av_ versus log(MW) for the known standards in part **c**; here K_av_ = (V_e_ – V_o_)/(V_t_ – V_o_) as described [2]. Based on empirical measurements of K_av_ for dmU1A and dmU1A(F37M/F77M), their molecular weights were estimated to be 12.6 kDa and 10.0 kDa (shown above); these values agree well with the true molecular weights of 11.4 kDa and 11.5 kDa for these samples.


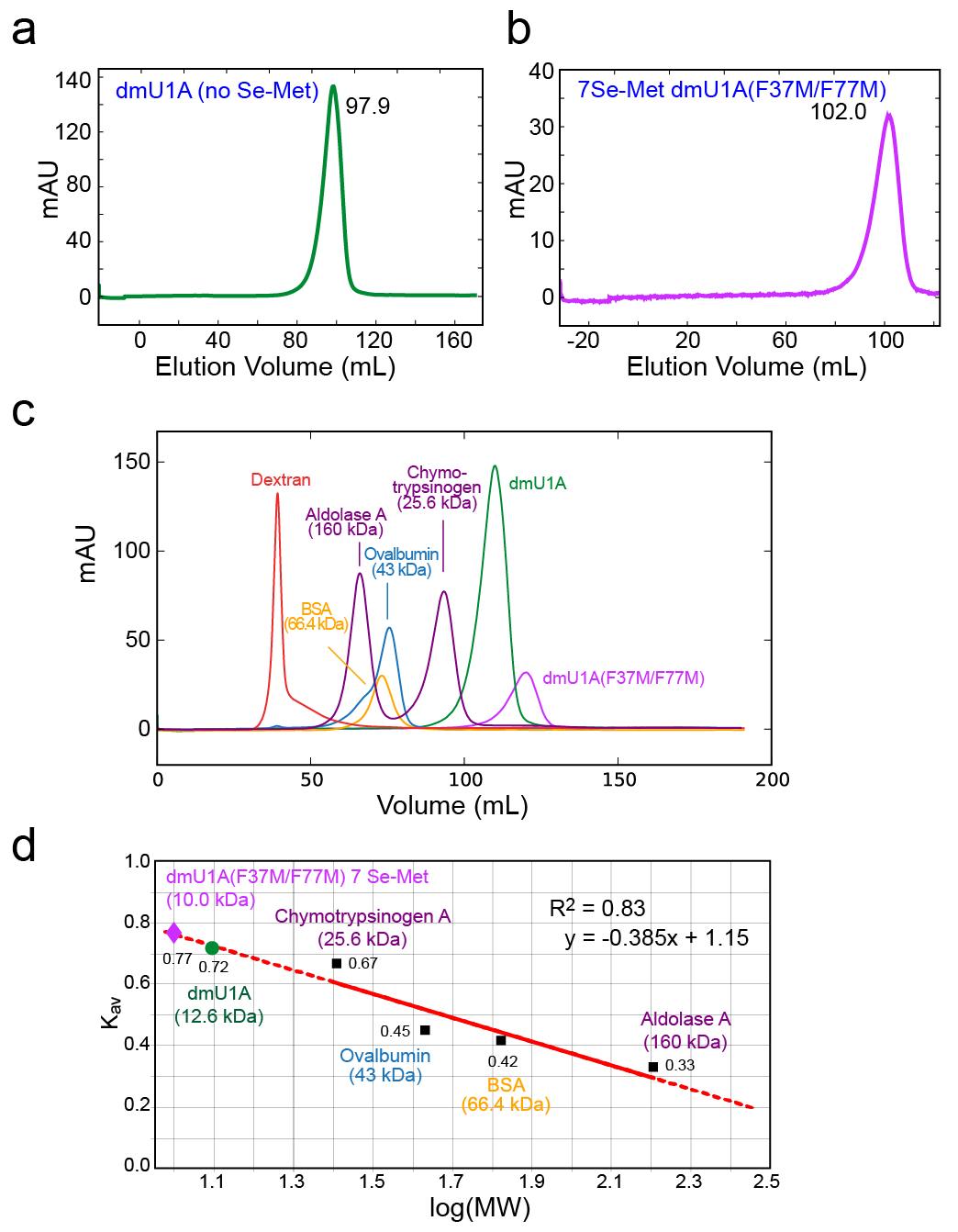


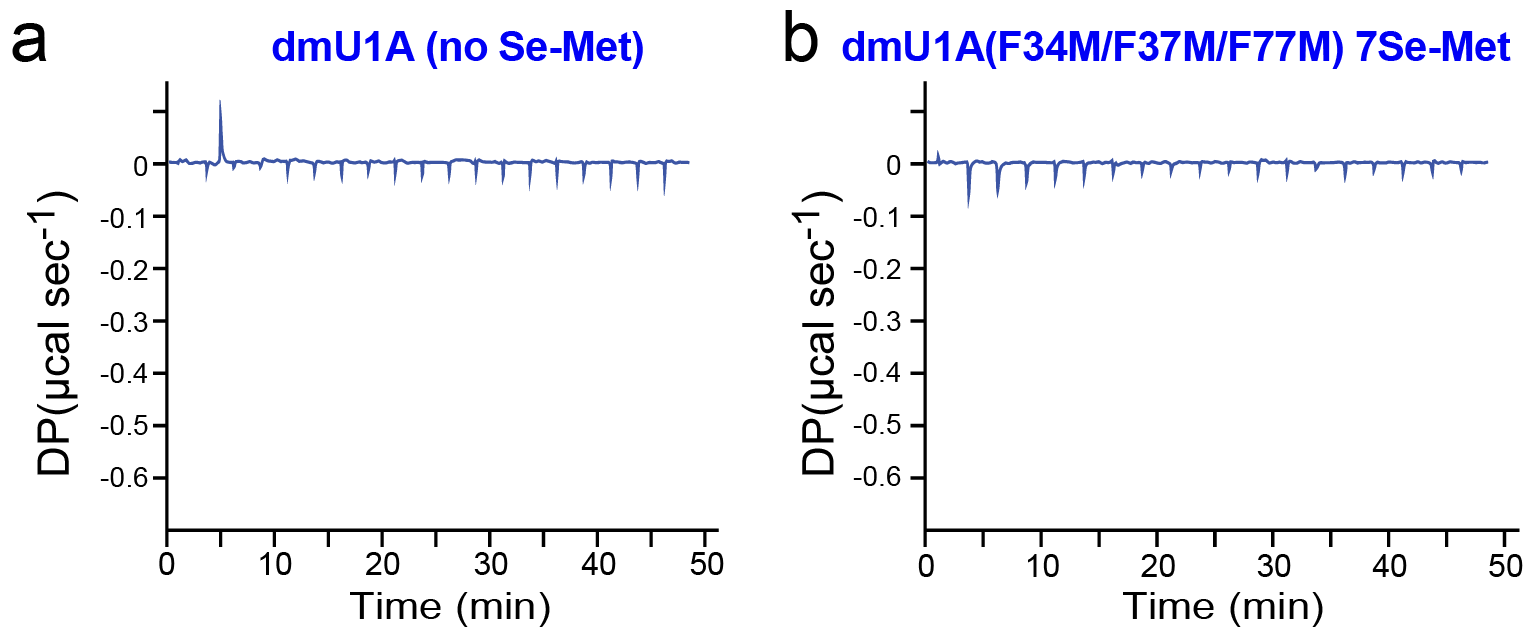


**Figure S2. ITC analysis of dmU1A and dmU1A(F37M/F77M) to assess heats of dilution.** (***a***) A titration of 150 μM dmU1A into buffer is shown. Here, the heat resulting from each injection of protein into RNA is comparable to the heat of dilution measured after saturation of hpII RNA with the titrated protein, as shown in **Figure 3b**. In contrast, the expected heat of dilution from a dimer-to-monomer transition is expected to be sigificantly larger [3]. (***b***) Τitration of 150 μM selenomethionine-labeled dmU1A(F37M/F77M) into buffer. The heat resulting from each injection of protein into RNA is comparable to the heat of dilution measured after saturation of hpII RNA with the titrated protein, as shown in **Figure 3d**. These observations demonstrate that the protein does not undergo an appreciable dimer-to-monomer transition that results from dilution during injection into the calorimeter sample cell.

| **Table S1: Intensity statistics for the Se-Met dmU1A(F37M/F77M)-preQ_1_-II riboswitch complex** | |
| --- | --- |
| **Data Collection** |  |
| Wavelength (Å) | 0.98 |
| Resolution range (Å) | 50.00 – 5.50 (5.59 – 5.50) |
| Space group | *F* 2 3 |
| a = b = c (Å) | 240.2 |
| α = β = γ (°) | 90.0 |
| Unique reflections | 12986 |
| Multiplicity | 3.5 (3.3) |
| Completeness | 97.6 (93.0) |
| Mean I / σ(I) | 10.7 (1.7) |
| *R*_sym_ | 11.6 (54.3) |

**References**

1. Appendix: Equations used for fitting ITC data, ITC data analysis in Origin. ITC Tutorial guide – version 07. **2004**, MicroCal ITC LLC, pp 104-113.

2. Scopes, R.K. in Protein Purification: Principles and Practice, 3^rd^ Edition. **1994**, Springer Verlag, New York. pp. 1-380.

3. Fernando, H.; Chin, C.; Rosgen, J.; Rajarathnam, K. Dimer dissociation is essential for interleukin-8 (il-8) binding to CXCR1 receptor. *J. Biol. Chem*. **2004**, 279, 36175-36178.
